# Proniosomal Gel for Topical Delivery of Rutin: Preparation, Physicochemical Characterization and In Vitro Toxicological Profile Using 3D Reconstructed Human Epidermis Tissue and 2D Cells

**DOI:** 10.3390/antiox10010085

**Published:** 2021-01-10

**Authors:** Iulia Pinzaru, Alina Tanase, Virgil Enatescu, Dorina Coricovac, Flavia Bociort, Iasmina Marcovici, Claudia Watz, Lavinia Vlaia, Codruta Soica, Cristina Dehelean

**Affiliations:** 1 Research Center for Pharmaco-Toxicological Evaluations, Faculty of Pharmacy, “Victor Babeș” University of Medicine and Pharmacy Timisoara, Eftimie Murgu Square No. 2, 300041 Timișoara, Romania; iuliapinzaru@umft.ro (I.P.); iasmina.marcovici@umft.ro (I.M.); farcas.claudia@umft.ro (C.W.); vlaia.lavinia@umft.ro (L.V.); codrutasoica@umft.ro (C.S.); cadehelean@umft.ro (C.D.); 2 Faculty of Pharmacy, “Victor Babeș” University of Medicine and Pharmacy Timisoara, Eftimie Murgu Square No. 2, 300041 Timișoara, Romania; 3 Faculty of Dental Medicine, “Victor Babeș” University of Medicine and Pharmacy Timisoara, 9 Revolutiei Bv., Sq., 300041 Timișoara, Romania; tanase.alina@umft.ro; 4 Faculty of Medicine, “Victor Babeș” University of Medicine and Pharmacy Timisoara, Eftimie Murgu Square No. 2, 300041 Timișoara, Romania; flaviabociort11@gmail.com

**Keywords:** rutin, antioxidant, proniosomal gel, 3D EpiDerm tissues, melanoma, keratinocytes

## Abstract

Rutin (Rut) is a natural flavonol, well-known for its broad-spectrum of therapeutic effects, including antioxidant and antitumoral activities; still, it has a reduced clinical outcome due to its limited solubility in aqueous solutions. To overcome this drawback, this study proposes a novel formulation for rutin as a proniosomal gel for cutaneous applications. The gel was prepared by coacervation phase-separation method and complies with the standard requirements in terms of particle size (140.5 ± 2.56 nm), zeta potential (−27.33 ± 0.09 mV), encapsulation capacity (> 50%), pH (7.002 ± 0.18) and rheological properties. The results showed high biocompatibility of the gel on the 3D reconstructed human epidermis model characterized by increased viability of the cells and a lack of irritant and phototoxic potential. The evaluations on 2D cells confirm the preferential cytotoxic effect of Rut on melanoma cells (IC_50_ value = 8.601 µM, nuclear fragmentation) compared to normal keratinocytes. Our data suggest that the proniosomal gel is a promising drug carrier for Rut in the management and prevention of skin disorders.

## 1. Introduction

In recent years it became clearer the fundamental role of oxidative stress in the development of different chronic maladies such as neurodegenerative diseases, cardiovascular pathologies, diabetes, aging, and cancer, leading to an increased interest in compounds with antioxidant properties [1]. Oxidative stress is defined as a disequilibrium between the oxidative events within the cells and their antioxidant defense responses, resulting in the damage of the cellular structures and disease development [2,3]. Skin is considered the largest barrier of the body and a target for oxidative stress induced by different external stressors (UV radiation, food additives, drugs, cosmetics, air pollutants, etc.) or endogenous factors (cytokines, growth factors, physiologic stimuli), effects that are directly correlated with various cutaneous disorders (atopic dermatitis, acne, scleroderma, photoaging, psoriasis, skin cancer, etc.) [4]. Reactive oxygen species (ROS) in the skin are generated mainly by keratinocytes and fibroblasts under specific conditions and can be both beneficial and harmful at this level [3,4]. UV-mediated ROS are commonly involved in the initiation of skin cancers [5]. Skin carcinoma represents one of the most common types of cancer worldwide and comprises nonmelanoma skin cancers (basal cell carcinoma—BCC and squamous cell carcinoma—SCC) and melanoma [6]. Even though BCC and SCC have a higher incidence as compared to melanoma, melanoma is responsible for most of the skin cancer-related deaths due to its metastatic behavior [7,8,9]. Another rare type of skin cancer known for its aggressiveness and for its high rate of lethality is Merkel cell carcinoma [10]. Skin disorders represent a considerable burden (medical and economic) both for patients and health care systems due to their high prevalence and incidence worldwide [11]. Thus, it became imperative to find effective compounds with the antioxidant potential to ensure ROS homeostasis and concurrently efficient skin protection and a lower risk to develop ROS-related illnesses.

A promising alternative for antioxidant agents is represented by the natural compounds. It has been amply documented that natural antioxidants can exert a protective effect against UV-induced skin cancer. Furthermore, melanomagenesis was shown to be greatly affected by compounds able to inhibit ROS generation [12].

**Figure 1:**
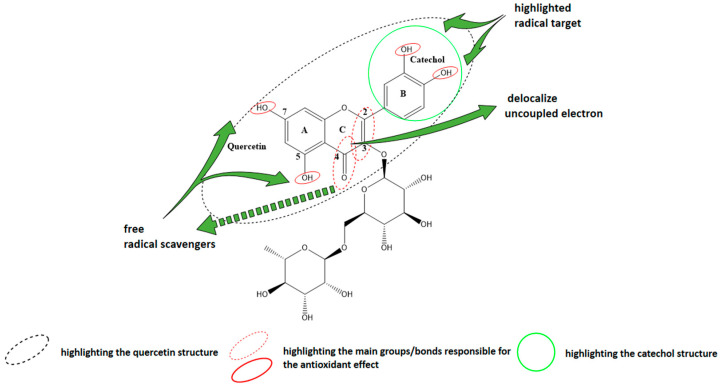
Chemical structure of rutin (Rut) highlighting the functional groups responsible for the antioxidant effect.

Rutin (IUPAC name 2-(3,4-dihydroxyphenyl)-5,7-dihydroxy-3-[(2S,3R,4S,5S,6R)-3,4,5-trihydroxy-6-[[(2R,3R,4R,5R,6S)-3,4,5-trihydroxy-6-methyloxan-2-yl]oxymethyl] oxan-2-yl]oxychromen-4-one) [13] is a quercetin rhamnoglucoside, also known as rutoside, sophorin, or quercetin-3-rutinoside, found in numerous plants, like: citrus, apples, vegetables, buckwheat, black tea, green tea, etc. [14] that falls into the profile of a promising antioxidant agent. It is a hydrophobic polyphenolic compound belonging to the family of flavonoids [15], one of the most valuable classes of plant-derived phytochemicals [16]. Due to its multispectral biochemical and pharmacological activities, especially antioxidant (as presented in Figure 1) and anti-inflammatory properties, this small molecule gained its place in various studies that aimed to assess its potential as an active ingredient [17,18,19,20,21] in pharmaceutical and cosmetic products [22].

Previous studies proved the beneficial effects of rutin in different skin disorders, like skin aging [23,24], psoriasis [25] and atopic and allergic contact dermatitis [26].

Besides the antioxidant and anti-inflammatory properties, rutin exerts multiple other pharmacological benefits, such as: cytoprotective, neuroprotective, vasoprotective, cardioprotective, analgesic and antiarthritic, antidiabetic, antihypercholesterolemic, antiplatelet aggregatory effect, antiulcer, antiasthmatic, antiosteoporotic and antiosteopenic, diuretic and anticancer properties [16].

A major drawback of rutin is represented by its low water solubility [22], leading to poor bioavailability and metabolic instability [15] and limiting its clinical use. Previous studies suggested that rutin in the form of nanoparticles or complexes exhibits desirable bioavailability [15]. In the last years, several studies presented different formulations of rutin to enhance its delivery via different routes, such as vesicular system based on a mixture of sorbitan monostearate and polyethylene glycol fatty acid esters (for rectal administration) [27], nanocrystals (SmartCrystals—to augment skin penetration) [28], rutin–phospholipid complex in polymer matrix [29], oil-in-water emulsions [30], rutin-loaded ethosomes (for topical use) [31], etc., but there is still room for improvements.

The drug delivery platforms that captured the attention of specialists in the pharmaceutical, medical, cosmetic, and nanotechnology fields are the vesicular drug delivery systems that include liposomes, niosomes, proniosomes, etc. [32]. The main benefit of liposomes is the permission to be used in medical preparations (recognized by EMA, FDA), which propelled them to the first position of formulations selected to provide protection for reactive or sensitive compounds against the light, humidity, pH, temperature, oxygen, etc. Among the advantages of liposomes as encapsulating agents for biologically active principles were stated: (i) increased efficacy and bioavailability; (ii) rapid elimination from the body due to a facile metabolism; (iii) attractive biological properties (low toxicity, biocompatibility, biodegradability), and (iv) ability to change surface area and size. The liposomes act via limiting the interaction between oxygen and the incorporated bioactive compound and slow release of the compound due to isolation in extramembrane environments [33]. Proniosomes represent an upgrade of both liposomes and niosomes, a provesicular carrier platform that ensures both an improvement regarding the physicochemical properties (longer stability, the convenience of transportation, distribution and storage), but also of pharmacological properties (longer presence in the systemic circulation, controlled release of the drug, augmented penetration to target organs via different routes of administration and reduced toxic effects) [32]. These vesicles can be formulated either as liquid crystals with jelly-like consistency or as free-flowing formulations and can encapsulate both hydrophobic and hydrophilic drugs [32], thus representing a promising candidate for drug delivery.

The aim of the current study was to develop and characterize (physicochemical properties and toxicological profile) a novel formulation of rutin for topical applications based on proniosomes. In addition, it was assessed the toxicological impact of rutin per se in healthy (human immortalized keratinocytes—HaCaT) and tumor (human melanoma cells—A375) cells in terms of cell viability and nuclear morphology.

## 2. Materials and Methods

### 2.1. Reagents and Cell Lines

Rutin (Rut), sorbitan monostearate (Span 60) and cholesterol were purchased from Sigma-Aldrich, Merck KgaA, soy lecithin (Lipoid S 75) from Lipoid GmbH (Ludwigshafen, Germany) and absolute ethanol from Merck (Darmstadt, Germany).

The human melanoma cell line—A375 (ATCC^®^ CRL-1619™) was acquired from American Type Culture Collection (ATCC) and the immortalized human keratinocytes—HaCaT (HaCaT-300493) from CLS Cell Lines Service GmbH. The specific cell culture medium—Dulbecco’s modified Eagle’s medium-high glucose (DMEM), penicillin–streptomycin mixture, phosphate saline buffer (PBS), trypsin-EDTA, Trypan blue, dimethyl sulfoxide (DMSO), and alamarBlue (resazurin) were provided by Sigma-Aldrich, Merck KgaA. The fetal calf serum (FCS) and Hoechst 33342 reagent (Invitrogen) were acquired from Thermo Fisher Scientific, Inc., Waltham, MA, USA.

### 2.2. Preparation and Characterization of Proniosomal Gel Formulations

The medicated proniosomal gel was prepared by the coacervation phase-separation method, frequently used in practice [32]. The procedure was performed in dry wide-mouthed glass containers and involved the following steps: (i) weighing of the required amounts of lipophilic surfactant, soy lecithin, cholesterol; (ii) addition of bioactive compound (rutin) solubilized in absolute ethanol; (iii) sealing of glass containers to prevent the solvent loss; (iv) warming the obtained mixture in a water bath at 60–70 °C for about 5 min while shaking until all the solid components, especially surfactant and cholesterol dissolved completely; (v) addition of distilled water prewarmed at 60 °C (to obtain a clear liquid); (vi) continuation of mixture warming at the same temperature for about 3 min under moderate shaking, and (vii) cooldown of the liquid mixture at room temperature when it was converted to a white creamy proniosomal gel, that was stocked in the dark for characterization. The concentration of rutin in the final preparation was 0.3%, and the composition of the formulation is described in Table 1.

The pH of control (proniosomal gel without the bioactive compound) and medicated proniosomal gel formulations was measured at 25 ± 2 °C by the compendial potentiometric method using a pH-meter (Sension™ 1 portable digital pH meter, Hach Company, Frederick, MD, USA). The following work protocol was used: 1 g of proniosomal gel was dispersed in 20 mL of distilled water by heating at 50 °C and stirring for 1 min. Then, the obtained dispersion was cooled and filtered. The pH was determined in the filtrate in triplicate.

Rheological studies were conducted to determine the flow behavior, viscosity, and consistency of the two proniosomal gels (control and medicated). The flow behavior and viscosity were assessed in steady-state flow mode of a stress-controlled rheometer (RheoStress 1, HAAKE, Vreden, France) equipped with a cone-plate geometry (1/60), and data analysis was performed using HAAKE RheoWin 4 version 4.30 software. The consistency of proniosomal formulations in terms of penetration degree and spreadability was measured by the compendial penetrometric method and the parallel-plate method, respectively. A penetrometer (PNR 12, Petrolab, Speyer, Germany) equipped with a micro-cone and suitable container, and the del Pozo Ojeda-Suñé Arbussá extensometer were used; the procedures described in the literature were followed [34,35]. Each rheological experiment was carried out in triplicate at 25 ± 2 °C.

Dynamic light scattering (DLS) and zeta-potential quantification were performed on a Zetasizer (Nano ZS system, Malvern Instruments, Malvern, UK); zeta-potential values were measured at room temperature, the stock solutions being diluted with Milli-Q water.

The encapsulation efficiency (EE) was conducted as described in the literature [36]. Briefly, the liposomes were subjected to destruction in order to obtain a more accurate evaluation and analysis of the encapsulated substance. The measurements were performed after obtaining the calibration curve (by using six concentrations of rutin, between 1 and 25 µM), and the absorbance reading at 373 nm (at room temperature) was conducted with a T70 UV-vis spectrophotometer (PG Instruments Ltd., Lutterworth, UK).

### 2.3. EpiDerm Testing

EpiDerm skin model (EPI-200) (Lot 30,838) and EpiDerm skin irritation model (EPI-200-SIT) (Lot 30838) were purchased from MatTek Corporation. Negative control (NC) for EPI-200 was ultrapure water (in the case of phototoxicity testing sesame oil depending on the lipophilic affinity of the main vehicle) and for EPI-200-SIT was Dulbecco’s phosphate-buffered saline (DPBS) while positive control (PC) for EPI-200 was Triton X-100 1% (provided by the manufacturer—MatTek Corporation, Ashland, MA, USA) and for EPI-200-SIT was sodium dodecyl sulfate (SDS) 1%. The specific media such as assay medium and Dulbecco’s phosphate-buffered saline (DPBS) rinse solution needed for the industrial model, assay medium (EPI-100-NMM) needed for the skin irritation model, EPI-200-PHO kit (serum-free assay medium (DMEM based) and Dulbecco’s phosphate-buffered saline (DPBS) rinse solution needed for the phototoxicity assay) were supplied by the manufacturer (MatTek Corporation).

The EPI-200 (EpiDermTM Skin Model) model was prepared as indicated in the MatTek protocol [37]. Briefly, the steps were as follows: (i) upon receipt, the inserts containing the tissues were removed from the agarose and transferred for 4 h in 0.9 mL/well of assay medium in a 6 well-plate at 37 °C and 5% CO_2_; (ii) following the 4 h incubation period, the assay media contained within the 6-well plates was replaced with 0.9 mL/well of prewarmed, fresh assay media and the tissues were further incubated overnight; (iii) the second day, the assay medium was renewed, and the test samples were added on the top of the inserts containing the tissues (25 µL/insert of DPBS to moisten the tissue surface followed by application of 100 mg/insert of the tested sample) and incubated for 18 h; (iv) the PC, Triton X-100 1% and the NC, ultrapure water, were also applied on the top of the inserts containing the tissues and incubated for 18 h at 37 °C and 5% CO_2_; (v) MTT assay. After the 18 h exposure period, the inserts were washed with DPBS and transferred in a 24-well plate containing 300 µL/well of 3-(4,5-dimethylthiazol-2-yl)-2,5-diphenyltetrazolium bromide (MTT—1 mg/mL) and incubated for 3 h. After the incubation period, the inserts were removed and placed into a new 24-well plate where each insert was immersed with 2 mL of the extractant solution (isopropanol-analytical grade). Then the extraction plate was sealed with parafilm and placed on a shaker for 2 h at room temperature (protected from light). After the extraction period has completed, 200 µL/well of the extraction solution was added into a 96-well plate, and the absorbance was spectrophotometrically measured at 570 and 650 nm, using a microplate reader (xMark microplate spectrophotometer, Bio-Rad, Tokyo, Japan). The data were corrected according to isopropanol, the solution used for the extraction of formazan crystals.

The viability of each of the tested samples was calculated using the following formula:

(1) $$\mathrm{viability}\%\;=\frac{{\mathrm{OD}}_{\mathrm{sample}}}{{\mathrm{OD}}_{\mathrm{NC}}}\;\times \;100$$

where: OD = optical density; NC = negative control.

#### 2.3.1. Reconstructed Human EpiDermal Model (EPI-200-SIT)—Skin Irritation Assay

The EPI-200-SIT model was prepared following the manufacturer’s instructions and data from specific literature [38,39]. The same five steps that were followed in the case of EPI-200, with the following specifications: step (i) incubation period 1 h; step (iv) PC, 1% SDS in saline and the NC, sterile DPBS; step (v) MTT assay was performed as described for EPI-200 (with the specification that the absorbance reading was done at 570 nm). The tissue viability (%) was calculated using the following formulas [38]:

(2) $$\mathrm{Relative}\;\mathrm{viability}\;\mathrm{TS}\%=\frac{{\mathrm{OD}}_{\mathrm{TS}}}{{\mathrm{Mean}\;\mathrm{of}\;\mathrm{OD}}_{\mathrm{NC}}}\;\times \;100$$

(3) $$\mathrm{Relative}\;\mathrm{viability}\;\mathrm{NC}\%=\frac{{\mathrm{OD}}_{\mathrm{NC}}}{{\mathrm{Mean}\;\mathrm{of}\;\mathrm{OD}}_{\mathrm{NC}}}\;\times \;100$$

(4) $$\mathrm{Relative}\;\mathrm{viability}\;\mathrm{PC}\%=\frac{{\mathrm{OD}}_{\mathrm{PC}}}{{\mathrm{Mean}\;\mathrm{of}\;\mathrm{OD}}_{\mathrm{NC}}}\;\times \;100$$

where: TS = test substance, NC = negative control, PC = positive control, OD = optical density.

#### 2.3.2. EpiDermTM Skin Model (EPI 200)—Phototoxicity Test

The phototoxicity protocol was realized in the same four steps that were followed in the case of EPI-200, with the following specifications: step (i) incubation period 1 h; step (iv) NC, sesame oil. In step (v), the plates covered with lids were exposed to ultraviolet A (UVA) irradiation (+UVA plates)—at 6 J/cm^2^ by means of the Biospectra system (Vilber Lourmat, France). In the meantime, the plates without exposure (−UVA plates) were kept in the dark at room temperature; after the UVA irradiation was completed, each insert was washed with DPBS. The same procedure was applied to the inserts that were not irradiated to avoid any variable conditions between the +UVA and −UVA plates. Then all inserts were transferred into new plates containing 0.9 mL/well assay medium and incubated for 18–24 h at 37 °C and 5% CO_2_. On the third day, the inserts were washed with DPBS and transferred in a 24-well plate containing 300 µL/well of MTT and analyzed in the same manner as for EPI-200-SIT.

The tissue viability was calculated using the following formulas [40,41]:

(5) $$\mathrm{RTV}\%=\frac{{\mathrm{OD}}_{\mathrm{sample}}}{{\mathrm{OD}}_{\mathrm{vehicle}\;\mathrm{control}}}\;\times \;100$$

(6) $$\mathrm{RTV}\;\mathrm{control}\%\;=\;\frac{{\mathrm{OD}}_{\mathrm{vehicle}\;\mathrm{control}}}{{\mathrm{OD}}_{\mathrm{mean}\;\mathrm{vehicle}\;\mathrm{control}}}\;\times \;100$$

where: RTV = relative tissue viability, OD = optical density.

### 2.4. Cytotoxicity on 2D Cell Culture

The in vitro cytotoxicity assessment was conducted on immortalized human keratinocytes—HaCaT and human melanoma cells—A375 using the alamarBlue assay. Cell culturing was performed according to the manufacturer’s instructions: cells were grown in specific culture media—DMEM high glucose completed with 10% fetal calf serum (FCS) and 1% antibiotic mixture (penicillin/streptomycin) solution and kept in standard conditions—an incubator with a humidified atmosphere (5% CO_2_) at 37 °C. In brief, the experimental protocol consisted of the following steps: (i) seeding of cells (A375 and HaCaT) in 96-well plates at a density of 1 × 10^4^ cells/200 µL medium/well and incubation until the proper confluence was reached; (ii) cells were treated with different concentrations of rutin (Rut) solution in DMSO (1, 2.5, 5, 7.5, 10, 20, 25, 50, and 75 µM) for a 24-h period; (iii) 20 µL of alamarBlue reagent was added in each well, and the plate was incubated for 3 h at 37 °C; (iv) the absorbance measurements were performed at 570 and 600 nm using an xMark™ microplate spectrophotometer (Bio-Rad), and (v) the results were expressed as the percentage of viable cells (%).

The impact of Rut on cell morphology was also monitored using the Olympus IX73 inverted microscope (Olympus, Tokyo, Tokyo, Japan). The images taken at 24 h posttreatment were analyzed using the cellSens Dimensions v.1.8. Software (Olympus).

### 2.5. Nuclear Staining

In order to verify the impact of rutin (5, 10, 20 and 50 µM) at nuclear level in healthy (HaCaT) and tumor (A375) cells after a 24 h treatment, it was performed the Hoechst 33342 staining assay. The protocol was applied as recommended by the manufacturer: (i) cell seeding (1 × 10^5^ cells/1.5 mL medium/well) in a 12-well plate; (ii) cell treatment with different concentrations of Rut in DMSO (5, 10, 20 and 50 µM) for 24 h; (iii) removal of old media (that contained test compound) after the treatment period and addition of 0.5 mL the staining solution (1:2000 in PBS) in each well; (iv) incubation for 10 min at room temperature and protected from light; (v) removal of the staining solution and washing 3 times with PBS, and (vi) taking pictures using Cytation 1 (BioTek Instruments Inc., Winooski, VT, USA). The analysis of the images was performed by means of Gen5™ microplate data collection and analysis software (BioTek Instruments Inc., Winooski, VT, USA). As a positive control for apoptosis induction, it was used 5 µM staurosporine (incubation for 3 h at 37 °C) and for necrosis 0.5% Triton X-100 (incubation for 30 min at 37 °C).

### 2.6. Statistical Analysis

The statistical data analysis was realized with GraphPad Prism version 8.3.0 for Windows, GraphPad Software, San Diego, California USA, www.GraphPad.com. The results are presented as the mean ± standard deviation (SD) by application of one-way ANOVA statistical test followed by Tukey’s post-test, and differences between the samples (control and treated) are marked with * (** *p* < 0.01, and **** *p* < 0.0001 vs. control cells/tissue).

## 3. Results

### 3.1. Preparation and Characterization of Medicated Proniosomal Gel

**Figure 2:**
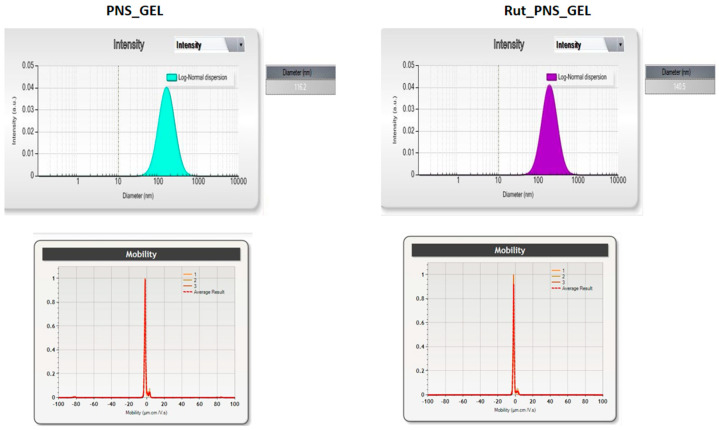
(**top**) Dynamic light scattering data for proniosomal control gel (PNS_GEL) and for proniosomal rutin-loaded gel (Rut_PNS_GEL); (**down**) mean sizes and distribution values for proniosomal control gel (PNS_GEL) and for proniosomal rutin-loaded gel (Rut_PNS_GEL).

**Figure 3:**
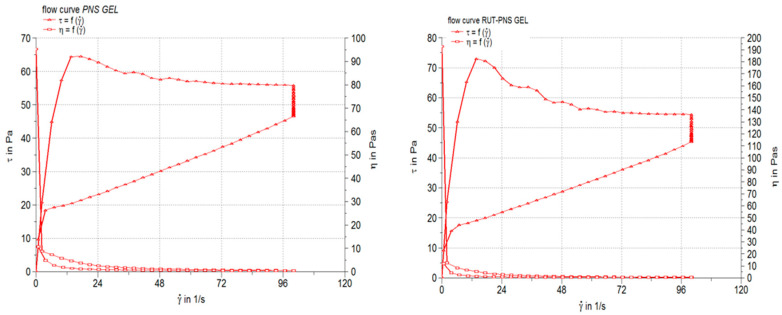
Rheograms and viscosity profiles of the proniosomal gels: control gel (**left**) and rutin-loaded gel (**right**).

**Figure 4:**
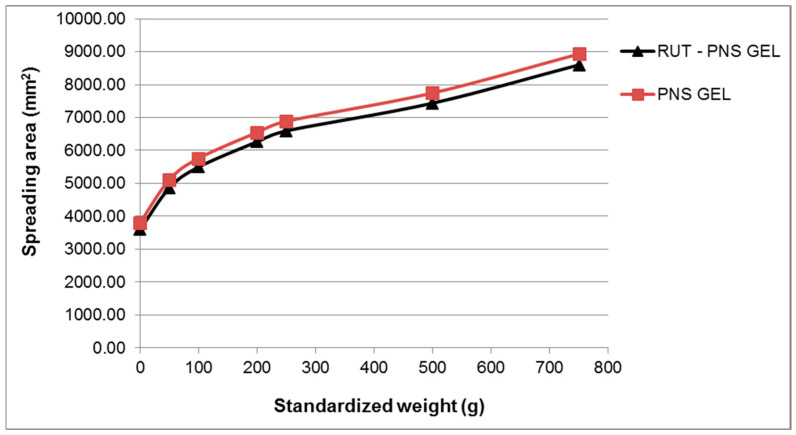
Extensiometric curves of experimental proniosomal gel containing 0.3% rutin and control formulation.

The specific characteristics of the obtained proniosomes (control and loaded with rutin) are presented in Figure 2, Figure 3 and Figure 4 and in Table 2. Proniosomes showed a negative zeta potential, and rutin-loading led to a slight change in the zeta potential to a more negative value (Figure 2). Regarding the average size and distribution of these types of biodegradable polymers, an increase in the size of proniosomes can be observed on a rutin basis compared to the size of control proniosomes. The polydispersity index was low, 0.15 for control proniosomes (PNS_GEL) and 0.19 for (Rut_PNS_GEL), confirming the homogeneous distribution and stability. The encapsulation efficiency is increased with a value exceeding 50%. The listed characteristics support the correct incorporation of the rutin into liposomes.

In Figure 3 are presented the results of the steady-state flow test, as rheograms (the shear stress, τ, as a function of shear rate, γ) and viscosity profiles (the apparent viscosity, η, as a function of shear rate). The pH value of the control proniosomal gel and rutin-loaded proniosomal formulation were neutral (7.105 ± 0.09 and 7.002 ± 0.18, respectively). Table 2 lists the apparent viscosity and thixotropy values obtained for the medicated proniosomal gel formulations and pH values.

The results of the spreadability test are depicted in Figure 4 as extensiometric profiles. The spreadability area increased progressively with the increasing of the applied weight, and for all the applied weights, the proniosomal gel containing 0.3% rutin showed larger spreading areas. The results of the consistency evaluation by penetrometry and spreadability test were in concordance with that obtained from the steady-state flow test, suggesting the influence of the chemical properties of incorporated drug molecules (rutin).

### 3.2. Toxicological Profile Evaluation of Rutin Proniosomal Gel in 3D Human EpiDerm Reconstructed Tissue

#### 3.2.1. Proniosomal Rutin Gel Showed a Proliferative Effect in 3D Human EpiDerm Reconstructed Tissue

**Figure 5:**
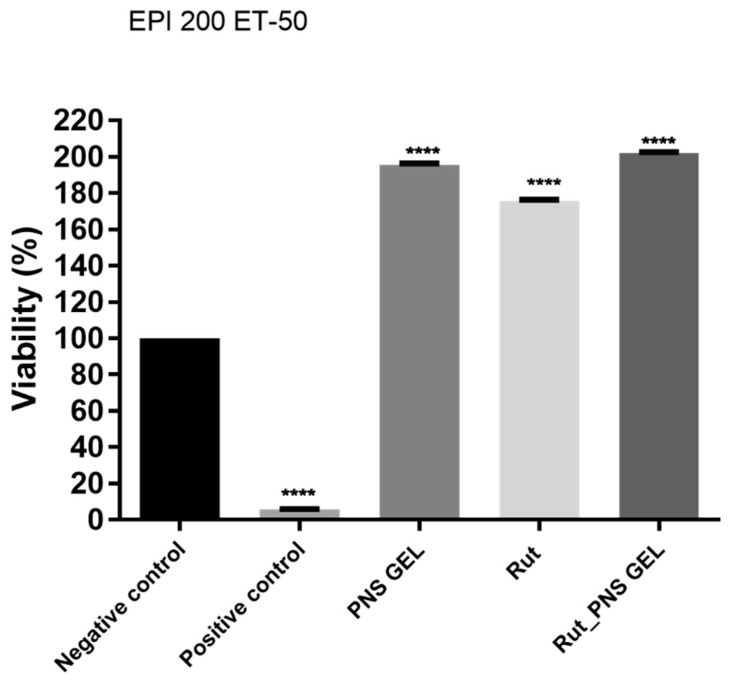
Viability percentage of EpiDerm skin model inserts (EPI 200 ET-50) at 18 h with the test samples (proniosomal control gel, rutin and proniosomal rutin gel). One-way analysis of variance (ANOVA) followed by Tukey’s post-test was applied to determine the statistical differences between sample-treated inserts vs. negative control-treated inserts (*p* < 0.0001 indicated by ****). Positive control is represented by Triton X-100 1%, and Milli-Q H_2_O was used as negative control.

Reconstructed human epiderm (RhE) viability was achieved using the MTT test after 18 h of exposure, and ultrapure water and Triton X-100 1% was used as negative control and positive control according to OECD recommendations. The viability of RhE after exposure to positive control and the incubation period of 18 h after application decreased to around 5% (Figure 5). Rutin and proniosomal gels (control and loaded with rutin) showed significantly higher viability than the positive control but also compared to the negative control. Rutin alone exerts a biological activity that increases cell viability up to 175.6%, while in the case of proniosomal rutin gel, the viability exceeds 200%.

#### 3.2.2. Proniosomal Rutin Gel Classifies as Non-Irritant to Skin

**Figure 6:**
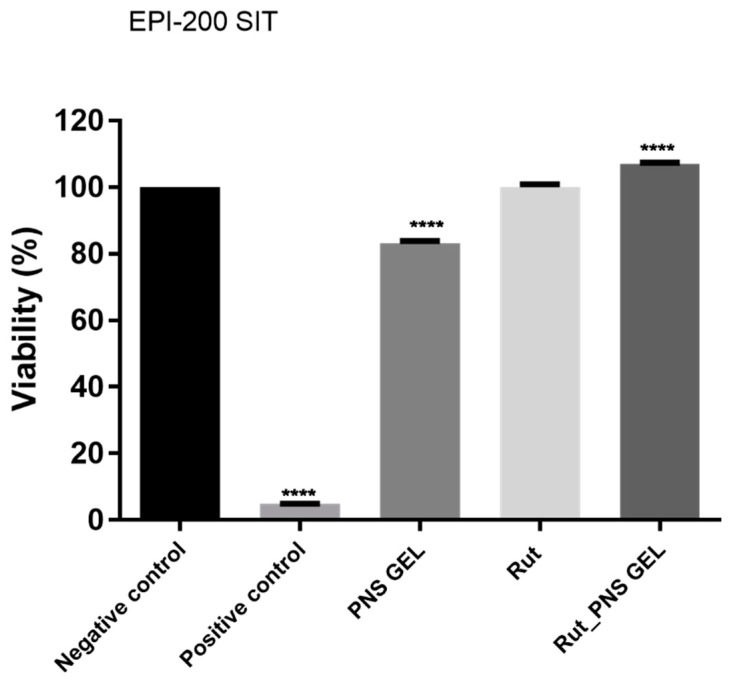
Viability percentage of EpiDerm skin model inserts (EPI-200 SIT) at 18 h posttreatment with the test samples (proniosomal control gel, rutin and proniosomal rutin gel). One-way analysis of variance (ANOVA) followed by Tukey’s post-test was employed to determine the statistical differences between sample-treated inserts and negative control-treated inserts (*p* < 0.0001 indicated by ****). Positive control is represented by SDS 1%, and negative control is represented by DPBS.

After the application of the standard protocol for skin irritation potential assessment, the test samples (proniosomal control gel, rutin and proniosomal gel) induced the following effects: a slight decrease in viability in the case of proniosomal control gel (83.2% viability), no influence on the viability in the case of rutin (100.1% viability) and a slight proliferative effect in the case of rutin proniosomal gel (~106.9% viability). Nevertheless, according to OECD Test Guideline 439, a sample is considered irritant if the viability of the sample-treated insert is below 50% after employing the skin irritation test. Regarding the irritant potential of the test samples (proniosomal control gel, rutin and proniosomal rutin gel), they did not show an irritant potential as the viability of each treated tissue exceeded 50% viability (Figure 6).

#### 3.2.3. Proniosomal Rutin Gel Showed a Non-Phototoxic Potential in 3D Human EpiDerm Reconstructed Tissue

**Figure 7:**
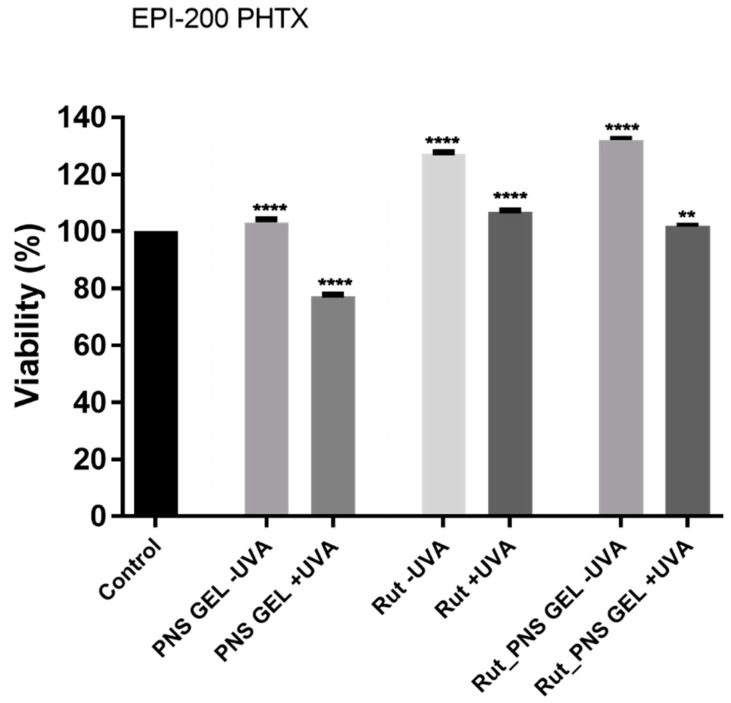
Viability percentage of EpiDerm skin model inserts (EPI-200 PHTX) at 24 h posttreatment with the test samples (proniosomal control gel, rutin and proniosomal rutin gel) ± UVA treatment. One-way analysis of variance (ANOVA) followed by Tukey’s post-test was employed to determine the statistical differences between sample-treated inserts and negative control-treated inserts (*p* < 0.01, *p* < 0.0001 indicated by ** and ****). Sesame oil or Milli-Q H_2_O was used as a negative control.

The results displayed in Figure 7 show the viability of EpiDerm inserts exposed to the test samples (proniosomal control gel, rutin and proniosomal rutin gel) with or without UVA exposure. According to the EpiDerm phototoxicity protocol [40,42], one sample is considered to have a phototoxic potential if it reduces the viability of the UVA-treated inserts by 25–30%, compared to the corresponding nonirradiated tissues. In the present study, only the proniosomal control gel sample could be attributed to elicit a slight phototoxic potential as the viability of the nonirradiated tissue stimulated with this one manifested a viability percentage of 103.2%, while the same sample induced viability of 77.3% on UVA treated tissues. At the same time, the proniosomal rutin gel sample induced a quite different result, showing an increased viability percentage (101.9%) on UVA irradiated tissues, whereas the UVA-free corresponding tissues manifested viability of 132.1%.

Among the above-mentioned samples, no significant viability differences between UVA-treated tissues and nonirradiated tissues were observed following exposure to rutin, which induced higher viability of EpiDerm inserts, 127.1% viability of UVA-free tissues and ~106.7% viability of UVA-treated inserts (Figure 7).

### 3.3. Cytotoxicity Assessment in 2D Cells

An in vitro cytotoxicity assessment was conducted to define the toxicological profile of Rut per se, using as experimental models the HaCaT (human immortalized keratinocytes) and A375 (human melanoma) cells. The evaluated toxicological parameters were cell viability, changes in cell morphology and nucleus aspect after a 24 h treatment with Rut solution in DMSO (1–75 µM).

#### 3.3.1. Rutin Showed a Non-Cytotoxic Effect in Human Immortalized Keratinocytes—HaCaT

**Figure 8:**
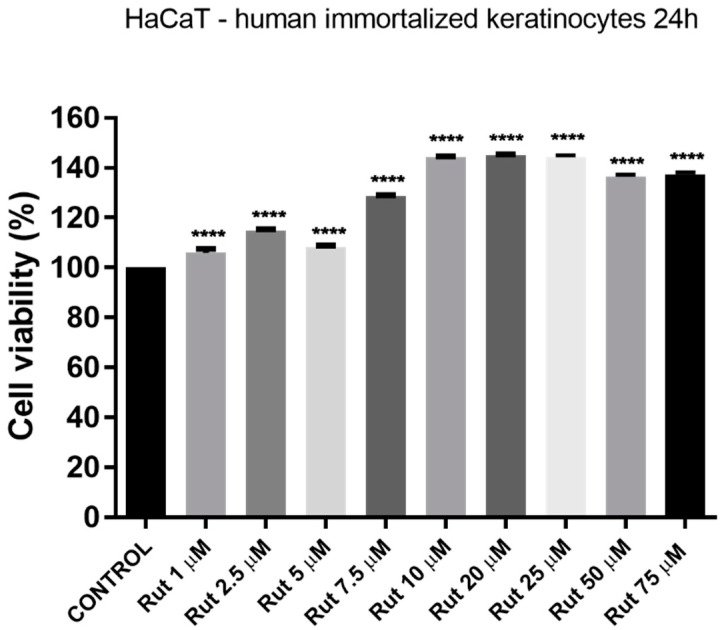
Evaluation of the in vitro effect of rutin (Rut; 1, 2.5, 5, 7.5, 10, 20, 50, and 75 µM) on human immortalized keratinocytes (HaCaT) viability after a 24 h treatment by applying the alamarBlue assay. The data are presented as cell viability percentage (%) normalized to control cells and expressed as mean values ± SD of three independent experiments performed in triplicate. Statistical differences between the control and the treated cells were calculated by one-way ANOVA analysis, followed by Tukey’s post-test (*p* < 0.0001 indicated by ****).

Treatment of HaCaT cells with different concentrations of Rut solution (1, 2.5, 5, 7.5, 10, 20, 25, 50, and 75 µM) for 24 h did not induce a decrease of cell viability. Instead, it was observed stimulation of cell viability percentage as compared to control cells (cells non-treated) (Figure 8). It was also verified the effect of DMSO (the solvent used for Rut solubilization) by testing the same concentrations as for Rut, and no significant differences were observed between control cells and the ones exposed to DMSO.

**Figure 9:**
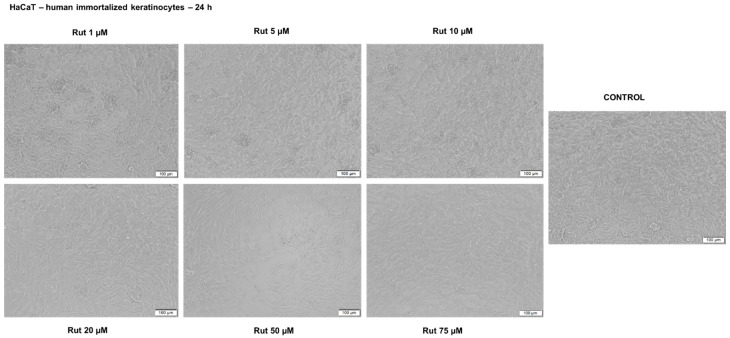
Morphological aspect of HaCaT cells after treatment with Rut (1, 5, 10, 20, 50 and 75 µM) for 24 h. The pictures were taken 24 h posttreatment. The scale bars represent 100 µm.

Another parameter that was evaluated in order to get a complete toxicological profile of Rut was its effect on HaCaT cell morphology, alterations in cell morphology being considered a sign of toxicity (Figure 9). As can be seen in the pictures below, Rut treatment did not affect the shape, and the adherence capacity of HaCaT cells as compared to control cells (non-treated cells) and no round and floating cells were observed. A slight decrease in confluency was observed at the highest concentrations of Rut (50 and 75 µM) (see Figure 9). As concerns the impact of DMSO on HaCaT cell morphology, there were no differences between the cells exposed to DMSO and the control cells.

**Figure 10:**
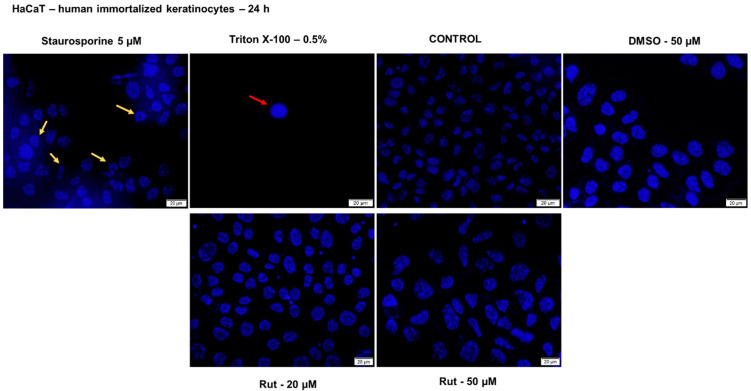
Cell nuclei staining using Hoechst 33342 in HaCaT cells after treatment with Rut (20 and 50 µM) and DMSO for 24 h. The pictures were taken 24 h posttreatment. Staurosporine solution (5 µM) represents the positive control for apoptotic changes at the nuclear level and Triton X-100 solution (0.5%) for necrosis. The yellow arrows indicate apoptotic cells with nuclear fragmentation, and the red arrow indicates a necrotic cell. The scale bars represent 20 µm.

Nuclear alterations following treatment with test compounds offer reliable data regarding the cytotoxic effect and indicate the cytotoxicity’s pattern (apoptosis or necrosis). On this basis, Hoechst 33342 staining assay was performed to verify the impact of Rut on HaCaT cells nuclei morphology after a 24 h treatment. As presented in Figure 10, the cell nuclei from the control group and DMSO-treated are round and regularly shaped, and no signs of nuclear fragmentation were noticed. A similar aspect was observed for the cells treated with Rut (for all concentrations tested), which indicates a lack of toxicity. Staurosporine solution (5 µM) was used as a positive control for apoptotic changes (chromatin condensation and nuclear fragmentation), and these changes are indicated by the yellow arrows. The HaCaT cells exposed to Triton X-100 solution (0.5%) presented a condensed and dense nucleus (red arrow), a specific sign for necrotic cells. The necrotic nuclear features (red arrows) induced by Triton X-100 solution were not detected in the HaCaT cells treated with Rut or DMSO. These data confirm the cell viability results indicating that Rut treatment has no cytotoxic effect on HaCaT cells at the tested concentrations.

#### 3.3.2. Rutin Induced a Dose-Dependent Cytotoxic Effect in A375 Cells

**Figure 11:**
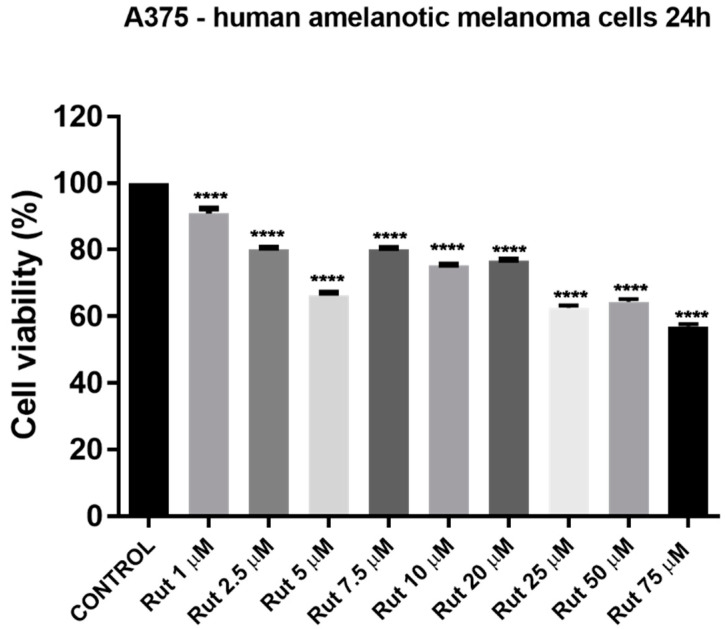
Evaluation of the in vitro effect of rutin (Rut; 1, 2.5, 5, 7.5, 10, 20, 50, and 75 µM) on human melanoma cells (A375) viability after a 24 h treatment by applying the alamarBlue assay. The data are presented as cell viability percentage (%) normalized to control cells and expressed as mean values ± SD of three independent experiments performed in triplicate. Statistical differences between the control and the treated cells were calculated by one-way ANOVA analysis, followed by Tukey’s post-test (*p* < 0.0001 indicated by ****).

Another cell line used in the present study as in vitro experimental model to establish the toxicological profile of Rut was A375—human melanoma cell line. For cell viability determination was conducted the same protocol as for HaCaT cells: treatment with different concentrations of Rut (1, 2.5, 5, 7.5, 10, 20, 25, 50, and 75 µM) for 24 h. The results indicated signs of cytotoxicity in A375 cells starting with the lowest concentration—1 µM, but the most significant decrease of the viable cells’ percentage was observed at the highest concentrations tested (25, 50 and 75 µM): 53.19%, 58.12% and 49.32%, respectively (see Figure 11). The calculated IC_50_ value for Rut is 8.601 µM. The solvent used for the preparation of the rutin stock solution (DMSO) manifested no effect in cancerous cell lines at the concentrations tested (the same as for the test compound).

**Figure 12:**
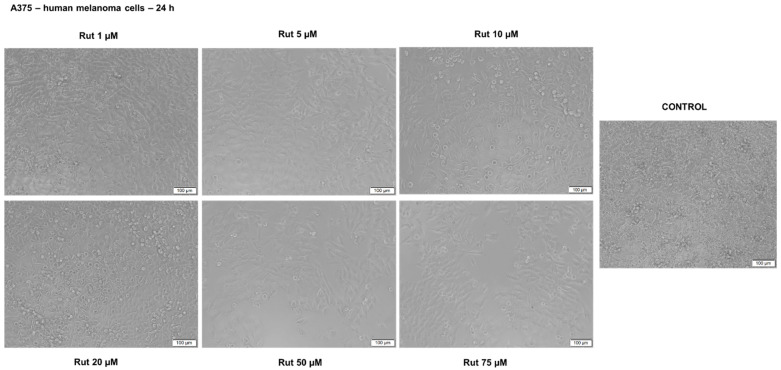
Morphological aspect of A375 cells after treatment with Rut (1, 5, 10, 20, 50 and 75 µM) for 24 h. The pictures were taken 24 h posttreatment. The scale bars represent 100 µm.

Rut treatment induced several changes in A375 cell morphology (Figure 12) as compared to control cells even at the lowest concentrations tested (1, 5, 10 and 20 µM) as round cells that were floating, whereas with increasing concentrations (50 and 75 µM) the number of round cells was increased, and a higher loss of adherence was detected together with a lower confluency, the cells being sporadically distributed on the plate. DMSO-treated cells presented no changes in terms of shape, adherence and confluency related to control cells. These data are consistent with the results obtained for cell viability evaluation.

**Figure 13:**
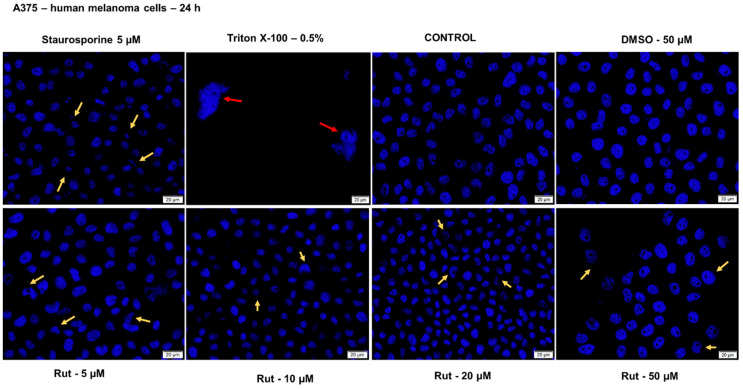
Cell nuclei staining using Hoechst 33342 in A375 cells after treatment with Rut (5, 10, 20 and 50 µM) and DMSO for 24 h. The pictures were taken 24 h posttreatment. Staurosporine solution (5 µM) represents the positive control for apoptotic changes at the nuclear level and Triton X-100 solution (0.5%) for necrosis. The yellow arrows indicate apoptotic cells with nuclear fragmentation, and the red arrow indicates necrotic cells. The scale bars represent 20 µm.

Taking into consideration the cell viability results that indicated a cytotoxic effect of Rut treatment on A375 cells, it was verified by means of Hoechst 33342 staining the pattern of cytotoxicity induced by Rut (Figure 13). Treatment with Rut (5, 10, 20 and 50 µM) induced nuclear fragmentation and chromatin condensation in A375 cells (yellow arrows) as compared to control and DMSO-treated cells (round nuclei with the regular shape) what indicates that cells undergo apoptosis. The nuclear changes observed in the case of A375 cells treated with Rut are similar to those induced by staurosporine solution (positive control for apoptosis—5 µM) but to a lower extent. The necrotic nuclear features (red arrows) induced by Triton X-100 solution were not detected in the A375 cells treated with Rut.

## 4. Discussion

The incisive climate changes recorded in recent years led to increased variability and aggression of the environmental factors and to a higher burden on skin barrier function. Human skin is a metabolically active organ with a protective role against environmental stressors (chemical factors, ultraviolet radiation, etc.) being compelled to permanently adapt its defense mechanisms [43]. The metabolic consequences of ultraviolet radiation (peroxidation of proteins and lipids, impairment of biological membranes, DNA mutations, development of cancer, etc.) are some of the most aggressive effects that occur at the cutaneous level, with clinical manifestations varying from inflammatory processes to melanoma [44]. Even though was observed an ascending trend for the use of skin protection products, there is a constant necessity for efficient products, antioxidant agents playing a key role in this context.

Antioxidant compounds that possess multiple phenol hydroxyl groups, such as rutin [16], are included in dermatological and cosmetic preparations as sun protection agents [45]. Despite the numerous studies conducted to produce, test and supply to consumers’ effective products, there is a significant gap of information regarding these products in terms of efficiency and safety-related issues.

The natural flavonoid, rutin, is considered an optimal protector of fibroblasts (acts against the alterations induced by ultraviolet radiation in the structure and functions of the phospholipid membrane) and a real candidate for dermatologic and cosmeceutical formulations, which presents a dual effect: a cytoprotective and effective treatment for different skin lesions (skin photoaging or premalignant and malignant lesions of the skin). Gegotek et al. concluded that ultraviolet radiation favorably affects the interactions between rutin and cell membrane: UVA facilitates membrane penetration through bilitranslocase and phospholipase A2 (PLA2) activity, and UVB contributes to the generation of ROS that produces membrane destabilization by increasing its permeability and thus facilitating interaction with phospholipids [46]. Rutin, in order to exert its effective biological action, must penetrate the cell membrane of fibroblasts. This target cannot be achieved without the development of a specific formulation that should have a dual role of protection and transport of the active substance. Various formulations have been suggested to improve rutin’s bioavailability and delivery [27,28,29,30,31]. Still, at present, it is not defined as a suitable formulation for rutin.

The present work was aimed to obtain a novel formulation of rutin for the topical application using as a drug delivery platform the proniosomes and to provide a complete characterization of the formulation in terms of physicochemical properties and toxicological profile.

The composition of the proniosomal gel used as a vehicle for rutin was selected based on the results of the previously published studies [47,48,49,50]. The proniosomal gel contained 0.3% (*w/w*) rutin and excipients, as described in Table 1. To avoid the potential hazards associated with certain components of the formulation were used only excipients that are recommended for such formulations and are considered safe. Liposomes are biodegradable materials that require increased wariness during preparation to avoid the release of chemicals harmful to the body [51].

Some of the limiting parameters in the case of medical applicability of liposome-based formulations are size and polydispersity index [52,53].

Conventionally, compounds with dimensions around 100 nm showed a longer half-life, and those with values in the range of 100–200 nm can penetrate the endothelial lining of blood vessels being directed much more easily to tumor cells compared to healthy ones, due to the good permeability and retention characteristics [54].

In this study, the proniosomal gel shows proper characteristics: particle size of 140.5 nm, a value that represents an adequate size for the release of an appropriate dose of the bioactive agent [52,55,56] and a ζ-potential of −27.33 mV. The zeta potential value obtained complies with the optimal stability range of 20–30 mV, considering the fact that at more positive or negative potential values, strong repulsions between particles leading to agglomeration take place [57]. The efficiency of encapsulation is influenced by the gel composition, which must be carefully selected, and the addition of cholesterol (not in excess to avoid competition for the encapsulation space required for the active substance) is recommended to increase the stiffness of the formulation to avoid loss of active substance [58,59].

The pH value of the rutin-loaded proniosomal gel was neutral (7.002 ± 0.18), complies with the recommended range for semisolid preparations (4.5–8.5) by the European Pharmacopoeia [34] and is close to the natural pH of the skin surface. Therefore, it can be suggested that the active formulation will be well tolerated by the skin. The obtained rheogram indicated that the prepared proniosomal gel is a non-Newtonian system with a pronounced pseudoplastic-thixotropic behavior but with a different degree of thixotropy. The pseudoplastic or shear-thinning behavior is highlighted by both rheograms profile, which is concave due to faster increase of the share rate than the shear stress, and viscosity curves indicating the viscosity decrease with the increase of the shear rate (Figure 3). The shear thinning-thixotropic behavior is an advantageous rheological property of medicated semisolid preparations, including proniosomal gels, because it is correlated with their ease of application and spreading on the skin. Changes in viscosity and thixotropy degree of studied proniosomal gel can be attributed to the chemical formula and properties of the incorporated drug (rutin), which affect the arrangement during the proniosomes formation process [60]. The viscosity values ranged in the specific field of topical semisolid preparations. The results of the consistency evaluation of the studied proniosomal gels through the penetrometric method are listed in Table 2 as penetration values. It can be observed that the formulation of RUT_PNS GEL produced a penetration value of 2.28-fold, indicating higher consistency for rutin-loaded formulations. The spreadability of the semisolid preparations, as rheological property related to consistency, is of interest because it contributes both to the application or delivery of a suitable drug dose on the skin and to the ease of semisolid application on the substrate. Moreover, the spreadability significantly influences the patient’s preference for the respective semisolid preparation [61].

Taking into consideration that topically applied compounds or formulations may have health consequences, international regulatory agencies (EMA, FDA) require an assessment of cutaneous safety for chemical compounds that are included in the final product for cosmetic, pharmaceutical and household use [62].

The cutaneous tests to verify the safety of the rutin-loaded proniosomal gel consisted of the evaluation of the gel’s impact on 3D human EpiDerm reconstructed tissues (MatTek) in terms of viability, skin irritation potential and phototoxicity.

Assessment of the irritant potential of a product (compound, formulation, etc.) is necessary to establish the ability to penetrate the *stratum corneum* (barrier) and to disrupt cellular integrity that could lead to local inflammatory processes. Skin damage can be caused by (a) a direct action (due to the physicochemical properties of the product) with the stabilization of lipid layers, the formation of pores and disruption of proteins in skin cells; and (b) an indirect action by releasing reactive oxygen species disrupting membranes and proteins in skin cells. These actions finally lead to cell death and the appearance of an inflammatory response at the tissue level clinically represented by redness, erythema, pain, edema, and itching [62]. The reconstituted human epidermis closely mimics the biochemical and physiological properties of the normal human epidermis and is constituted of human-derived keratinocytes (cultivated to achieve a multidimensional model, with several layers—basal, spinous, and granular, organized and a multilayered horny layer, composed of intercellular lamellar lipid layers) that are not transformed as a cellular source (having representative histology and cytoarchitecture) [63]. Specific skin irritation tests (SIT) are based on cellular behavior following the onset of skin irritation, cell death and inflammation and are used as particular biomarkers to quantify the potential of products/substances/compounds to cause skin irritation [39].

Topical exposure of the reconstructed human epidermis tissues to rutin samples (proniosomal gels and rutin), followed by cell viability analysis (by reducing MTT), showed a lack of toxicity and safety of use in terms of irritant potential. In our study, the IL-1 α analysis was not required due to the lack of irritant potential of the tested samples (as can be seen in Figure 6). Classification as irritant or non-irritant is based on the number of viable cells; a substance is considered to have irritant potential when less than half of the cells are viable at the end of the experiment (and non-irritant when more than half of the cells are viable at the end of the experiment) [62].

The EpiDerm phototoxicity test was used to evaluate the phototoxic effect, which is much more advantageous compared to the validated and regulated alternative that recommends the use of a 2D mouse fibroblast culture. In recent years, the European Federation of Pharmaceutical Industries and Associations and the European Medicines Agency has recognized the usefulness of reconstructed human epidermis for topical phototoxicity testing, implementing the protocol for testing the safety of dermally applied drugs [64,65]. The main advantages are those related to the similarity of the tissue in terms of the *stratum corneum* composition and lipid profile with the human epidermis while allowing direct testing of the actual formulations, which is not possible with correct and concrete results in the case of 2D cell cultures (lack of barrier function and solubility problems). For this reason, the samples (proniosomal control gel, rutin and proniosomal rutin gel) were tested on RhE.

Another aspect investigated in the present study was the potentially toxic impact of Rut (solubilized in DMSO) in human immortalized keratinocytes—HaCaT cells and human melanoma cells—A375. The HaCaT cell line was selected as an experimental model for healthy/normal cells based on the following considerations: (i) keratinocytes are located in the external skin layer and are the most exposed skin cells to UV noxious effects triggering changes in their cellular functions [44]; (ii) keratinocytes are the main sources for mitochondrial ROS at cutaneous level; (iii) HaCaT cells are immortalized cells that keep their morphological features during multiple passages in culture and offer reliable and reproducible results, and iv) these cells were also used in other studies for in vitro rutin evaluation [66].

Our results regarding the lack of a cytotoxic effect of Rut on HaCaT cell viability and morphology (Figure 8, Figure 9 and Figure 10) corroborate previously reported data: no cytotoxicity was induced even at concentrations as high as 1 mM Rut [66] (alone or in combination with ascorbic acid) against UVA and UVB radiation noxious effect [67,68].

A beneficial effect of rutin, not extensively investigated, is represented by the antitumoral activity. Treatment of A375 melanoma cells for 24 h with Rut (1–75 µM) determined a dose-dependent cytotoxic effect characterized by a decreased percentage of viable cells (IC_50_ = 8.601 µM, Figure 11), changes in cells morphology (round and floating cells, loss of adherence and reduced confluency—Figure 12) and nuclear alterations (nuclear fragmentation—Figure 13), a specific sign for apoptosis. Similar results were described by Khorsandi et al. The study investigated the cytotoxicity effect of rutoside (0, 5, 10, 25, 50, and 100 μg/mL) on A375 cell line after 4 and 24 h of stimulation. Their results revealed that the survival of melanoma cancer cells decreased in the presence of Rut and the cell viability was 54% and 43% at the concentration of 100 μg/mL after 4 h and 24 h, respectively. The calculated the IC_50_ value for Rut was approximately 100 µg/mL.

Another study evaluated the antimelanogenesis activity of the leaf extract of *Mallotus japonicus* and its main contributing compound (rutin) on B16F1 melanoma cells, as well as their cytotoxic activity. In the case of pigmented melanoma cells, a high survival rate was observed after the treatment with rutin for 24 h, suggesting its reduced anticancer activity on melanotic melanoma cells.

Previous studies presented the anticancer effect of rutin in different human cancer cells: lung cancer (A549), colon cancer (HT-29 and Caco-2) [20], leukemia (HL-60), neuroblastoma (LAN-5) [16] and melanoma (A375) [69]. The anticancer mechanism of action of Rut was inhibition of cancer cell proliferation, reduction of ROS production, suppression of cell migration and induction of apoptosis [20].

## 5. Conclusions

The present study showed that proniosomal gel based on biocompatible excipients (sorbitan monostearate, cholesterol and soy lecithin) was efficiently prepared by a simple method to serve as a drug carrier and vehicle for topical administration of rutin. The proniosomal formulations proved to be optimum in terms of particle size, zeta potential, drug encapsulation, pH and rheological properties. The safety profile of the proniosomal gels using 3D human reconstructed epidermis tissues indicated a lack of toxic effects after assessment of the viability, a skin irritant and phototoxicity potential. In addition, rutin proved to be toxic on A375 melanoma cells by inducing a reduction of cell viability, alteration of cell morphology and nuclear fragmentation, whereas in the case of human keratinocytes—HaCaT cells, no cytotoxic effect was noticed even at the highest concentration tested—75 µM. These data may serve as a promising alternative of drug delivery carrier and vehicle for topical use for chemical and natural compounds with limited solubility in water and in lipids.

## Figures and Tables

**Table 1:** The composition of medicated (rutin) proniosomal gel formulation.

Components	Composition
Span 60 (mg)	180
Cholesterol (mg)	30
Soy lecithin (mg)	90
Absolute ethanol (mL)	0.3
Distilled water (mL)	0.1
Bioactive compound (rutin) (%)	0.3

**Table 2:** Proniosomal gels characteristics: viscosity, thixotropy, penetration values and pH.

Gel Code	Viscosity (Pas)	Thixotropy (Pa/s)	Penetration Value (mm)	pH Value	Size (nm)	ζ Potential (mV)	EE (%)
PNS GEL	0.502 ± 0.24	2576	233.00 ± 1.15	7.105 ± 0.09	116.2 ± 1.13	25.53 ± 0.2	-
RUT_PNS GEL	0.488 ± 0.62	2930	249.00 ± 0.82	7.002 ± 0.18	140.5 ± 2.56	27.33 ± 0.09	59.6 ± 4.8

## Data Availability

Not applicable.
